# Farrerol alleviates insulin resistance and hepatic steatosis of metabolic associated fatty liver disease by targeting PTPN1

**DOI:** 10.1111/jcmm.70096

**Published:** 2024-09-17

**Authors:** Jingwen Gao, Xiaomin Cang, Lu Liu, Jiaxi Lin, Shiqi Zhu, Lihe Liu, Xiaolin Liu, Jinzhou Zhu, Chunfang Xu

**Affiliations:** ^1^ Department of Gastroenterology The First Affiliated Hospital of Soochow University Suzhou Jiangsu China; ^2^ Suzhou Clinical Center of Digestive Diseases Suzhou China; ^3^ Department of Endocrinology Affiliated Hospital 2 of Nantong University and First People's Hospital of Nantong City Nantong China

**Keywords:** farrerol, hepatic steatosis, insulin resistance, metabolic associated fatty liver disease, PTPN1

## Abstract

Metabolic associated fatty liver disease (MAFLD) is the most common chronic liver disease worldwide, characterized by excess lipid deposition. Insulin resistance (IR) serves as a fundamental pathogenic factor in MAFLD. However, currently, there are no approved specific agents for its treatment. Farrerol, a novel compound with antioxidant and anti‐inflammatory effects, has garnered significant attention in recent years due to its hepatoprotective properties. Despite this, the precise underlying mechanisms of action remain unclear. In this study, a network pharmacology approach predicted protein tyrosine phosphatase non‐receptor type 1 (PTPN1) as a potential target for farrerol's action in the liver. Subsequently, the administration of farrerol improved insulin sensitivity and glucose tolerance in MAFLD mice. Furthermore, farrerol alleviated lipid accumulation by binding to PTPN1 and reducing the dephosphorylation of the insulin receptor (INSR) in HepG2 cells and MAFLD mice. Thus, the phosphoinositide 3‐kinase/serine/threonine‐protein kinases (PI3K/AKT) signalling pathway was active, leading to downstream protein reduction. Overall, the study demonstrates that farrerol alleviates insulin resistance and hepatic steatosis of MAFLD by targeting PTPN1.

## 1 INTRODUCTION

Metabolic associated fatty liver disease (MAFLD) has emerged as the most common liver disease and plays a prominent role in contributing to liver‐related morbidity and mortality rates.^1^ This multifaceted spectrum of liver disorders encompasses steatosis, characterized by the accumulation of fat in the liver, with some recent studies shedding light on the molecular mechanisms involved. For instance, it has been demonstrated that protein tyrosine phosphatase non‐receptor type 1 (PTPN1),^2^ patatin‐like phospholipase domain‐containing protein 3 (PNPLA3),^3^ transmembrane 6 superfamily member 2 (TM6SF2)^4^ and et al are involved in lipid accumulation, providing further insights into the complex nature of this condition. Nonetheless, these disorders can progress to more severe conditions such as steatohepatitis and liver fibrosis.^5^, ^6^ Despite our growing understanding of the disease, the lack of approved pharmacological interventions for MAFLD remains a significant concern in the medical community. Current attempts to mitigate the complications associated with this condition have proven to be unsatisfactory.^7^ Consequently, there is an urgent need to develop effective therapeutic approaches to address this highly prevalent disease.

Insulin resistance (IR) is a metabolic abnormality often observed in subjects with MAFLD, and it has been considered a major determinant in the pathogenesis of metabolic dysfunction associated steatohepatitis (MASH) and the progression of liver disease.^8^, ^9^ PTPN1, also known as protein tyrosine phosphatase 1B (PTP1B), belongs to the protein tyrosine phosphatase (PTP) family.^10^ PTPs are a group of proteins that possess a significant role in negatively modulating the insulin signalling cascade by binding to and dephosphorylating critical components of the insulin pathway, including the insulin receptor (INSR), as well as insulin receptor substrate (IRS) 1 and 2, leading to their inactivation.^11^, ^12^ In addition, recent studies have shown that increased PTPN1 is involved in tumour immunity and promotes tumour growth.^13^, ^14^ Consequently, PTPN1 inhibitors are potential candidates for the treatment of metabolic diseases, including but not limited to fatty liver, obesity, type 2 diabetes and cardiovascular disease.^15^, ^16^

Farrerol, a novel flavonoid compound derived from the traditional Chinese medicinal herb ‘Man‐shan‐hong’ (the dried leaves of *Rhododendron dauricum* L., Ericaceae),^17^ has gained popularity for its therapeutic applications.^18^, ^19^ Previous studies have reported various pharmacological activities of farrerol, including antioxidant, anti‐inflammatory and antibacterial effects.^20^, ^21^, ^22^ Recent investigations have highlighted its protective role against acetaminophen‐induced liver injury by modulating autophagy.^23^ Additionally, farrerol can ameliorate streptozotocin‐induced hyperglycemia and dyslipidemia via the attenuation of oxidative stress and inflammation.^24^ However, the potential therapeutic effects of farrerol on MAFLD and its underlying mechanism remain unclear.

The primary objective of this study was to investigate the impact of farrerol on the pathogenesis of MAFLD, with a specific focus on its effects on lipid accumulation. Mechanistically, our findings elucidated farrerol as a potential therapeutic candidate for treating MAFLD, as it exerted its effects through the direct inhibition of PTPN1.

## 2 MATERIALS AND METHODS

### 2.1 Database introduction and data mining

The PubChem database (https://pubchem.ncbi.nlm.nih.gov/), specifically the compounds’ module, is a part of the National Center for Biotechnology Information (NCBI). Within PubChem, the compounds module provides extensive information on chemical compounds, including detailed structures, properties, activities and references.

Integrated Traditional Chinese Medicine (ITCM) integrates 10 TCM‐related databases, including 25,857 formulas, 8454 herbs, 43,430 ingredients, 18,851 targets, 11,180 diseases and 1488 profiles (http://itcm.biotcm.net/).

TargetNet is a database that analyses the chemical features and structural properties of compounds for predicting potential protein targets (http://targetnet.scbdd.com).^25^

SwissTargetPrediction (SwissTarget) is a platform that enables users to predict the potential macromolecular targets of small molecules. It utilizes a combination of 2D and 3D similarity analysis with a library of over 370,000 known active compounds across over 3000 proteins from three species (http://swisstargetprediction.ch/).^26^

RCSB Protein Data Bank (RCSB PDB) offers powerful search and analysis tools for exploring protein structures and visualizing molecular details. The Protein‐Ligand Interaction Profiler is a tool to identify non‐covalent interactions between biological macromolecules and their ligands easily (https://www.rcsb.org/).^27^

DisGeNET is a comprehensive and freely accessible database that focused on exploring gene‐disease associations, enabling users to retrieve relevant data, visualize gene‐disease networks and prioritize candidate genes for further investigation (https://www.disgenet.org/home/).^28^, ^29^, ^30^

GPSAdb is a comprehensive web resource providing interactive exploration of genetic perturbation RNA‐seq datasets (https://www.gpsadb.com/).^31^ Users can identify candidate causal perturbations from differential gene expression data.

STRING (Version 12.0) is a widely used bioinformatics database providing comprehensive information on protein–protein interactions (PPIs). It integrates and analyses data from multiple sources to create a network of functional associations between proteins (https://string‐db.org/).

### 2.2 Kyoto Encyclopedia of Genes and Genomes (KEGG) pathway enrichment analysis

To identify the underlying biological processes affected by the differentially expressed genes, we performed KEGG pathway enrichment analysis using the clusterProfiler R package (Rtools4.3). The statistical significance was assessed using the hypergeometric test, and pathway enrichment analysis revealed significant enrichment (adjusted *p*‐value <0.05) in several KEGG pathways.

### 2.3 Molecular docking

Molecular docking simulations were performed using OpenBabel‐3.1.3, PyMOL‐2.3.4 and AutoDock Vina.^32^ The crystal structure of PTPN1 was obtained from the Protein Data Bank (PDB ID: 5QF5), and the ligands of interest were prepared by SailVina. The protein structure was processed by removing water molecules. Docking grids were defined around the active site based on the known binding pocket residues.

### 2.4 Animals and treatments

Healthy 5‐ to 6‐week‐old male C57BL/6J mice were housed in cages under standard laboratory conditions (at a temperature of 22–24°C temperature, 55%–60% relative humidity and a 12 h/12 h light/dark cycle). A mouse model of NASH was established by feeding the mice an high‐fat diet (HFD) free of trans fats (D09100301; 40% kcal of fat, 40% kcal of carbohydrates, 20% kcal of protein, 2% mass of cholesterol; Research Diets, Wuxi, China) for 16 weeks. Mice fed a normal diet (ND) (10% fat, 70% carbohydrate and 20% protein; Trophic Diets, Suzhou, China) served as controls. Farrerol (40 mg/kg) (HY‐N0344, MedChemEpress, USA) was administered daily by intraperitoneal (i.p.) injection to the model mice for 8 weeks.^23^

### 2.5 Cell culture and stimulus

The hepatocyte cell line HepG2 was purchased from Shanghai FuHeng Biology Technology Co., Ltd. (CL‐D103, China). Cells were cultured in Dulbecco's modified Eagle's medium (DMEM) (c11995500BT, Gibco, USA) with 10% foetal bovine serum (S711‐001S, LONSERA, China) and placed in 5% CO_2_ under a humidified atmosphere at 37°C. HepG2 cells were stimulated with a 1.0 mM palmitic acid (P0500, Sigma‐Aldrich, USA)–oleic acid (O1008, Sigma‐Aldrich, USA) (PAOA) mixture (PA:OA = 1:2) in the medium for 24 h to establish the cellular lipid accumulation model. Farrerol (20 μM) was then administered for 24 h.^23^ When insulin signalling was measured, the cells were stimulated with 100 nmol/L insulin for 10 min and then collected for western blot.^33^

For adenoviral infections, cells were grown to 30%–40% confluence and changed to a serum‐free medium containing recombinant adenoviruses expressing the green fluorescent protein (Ad‐GFP) or Ad‐PTPN1. The infected cells were incubated for 1.5 h and then restored to normal DMEM containing 10% foetal bovine serum for an additional 2 days of incubation.

### 2.6 Glucose tolerance test (GTT), insulin tolerance test (ITT) and pyruvate tolerance test (PTT)

Prior to the GTT experiment, animals underwent a fasting period of 6 h. After fasting, the animals were administered an i.p. injection of glucose based on their body weight (0.75 g/kg body weight). For ITT, the mice fasted for the same amount of time and were then administered an i.p. injection of insulin (1.5 U/kg body weight). For PTT, the mice fasted for the same amount of time and were then administered an i.p. injection of pyruvate (2 g/kg body weight). Blood glucose concentrations were measured at 0, 15, 30, 60, 90 and 120 min after injection.^34^, ^35^ The homoeostatic model assessment (HOMA) is a method used to quantify insulin resistance (HOMA‐IR). HOMA‐IR = (Insulin × Glucose) /22.5. Insulin concentration is reported in μU/L (1 μU/mL of insulin is equivalent to about 0.0347 μg/L) and glucose in mmol/L. The constant of 22.5 is a normalizing factor.^36^

### 2.7 Histological analysis

A part of the liver was isolated, fixed in a 4% formaldehyde solution and embedded in paraffin wax. Subsequently, 3 μm sections were cut and stained with haematoxylin and eosin (BA4025A, Baso, China) and periodic acid–Schiff (P0430, Sigma‐Aldrich, USA). Another part of the liver was dehydrated with sucrose and embedded in OCT. Ten micrometre frozen sections were then stained with Oil Red O (C0158M, Beyotime, China). HepG2 cells were fixed in a 4% formaldehyde solution and then stained with Oil Red O.

### 2.8 Determination of liver biochemical parameters

The serum were collected from mice after a 6‐hour fasting period. The serum levels of triglycerides (TGs), total cholesterol (TC), high density lipoprotein (HDL) and low density lipoprotein (LDL) and the activity of the liver‐associated enzymes alanine aminotransferase (ALT) and aspartate aminotransferase (AST) were estimated using a BS‐850 chemistry system analyzer (Mindray, China) according to the manufacturer's instructions.

### 2.9 Western blot assay

Total protein was isolated from liver tissue and HepG2 cells using RIPA lysis buffer (R0278, Sigma‐Aldrich, USA) mixed with a protease inhibitor (HY‐K0021, MedChemExpress, USA). A BCA protein assay kit (BL521A, Biosharp, China) was used to determine the protein concentration. The protein samples were separated using a 10% sodium dodecyl sulphate‐polyacrylamide gel and transferred onto PVDF membranes (1620177, Bio‐Rad, USA). Blots were probed with primary antibodies against INSR (sc‐57344, Santa, USA), phospho‐INSR (p‐INSR) (sc‐534471, Santa, USA), phosphatidylinositol‐4,5‐bisphosphate 3‐kinase catalytic subunit alpha (PIK3CA/PI3K) (ab191606, Abcam, UK), phospho‐PI3K (p‐PI3K) (#17366, Cell Signaling, USA), AKT, phospho‐AKT (p‐AKT) (ab283852, Abcam, UK), PTPN1 (11334‐1‐AP, Proteintech, China), sterol regulatory element binding transcription factor 1 (SREBF1/ Sterol Regulatory Element‐Binding Protein 1 (SREBP1)) (66875‐1‐AP, Proteintech, China), stearoyl‐CoA desaturase (SCD) (28678‐1‐AP, Proteintech, China), peroxisome proliferator activated receptor Gamma (PPARG/PPARγ) (16643‐1‐AP, Proteintech, China). Next, horseradish peroxidase (HRP)‐conjugated goat anti‐rabbit (SA00001‐2, Proteintech, China) or anti‐mouse (SA00001‐1, Proteintech, China) secondary were applied. Finally, the proteins were detected by enhanced chemiluminescence (WBKLS0500, Merck KGaA, Germany). The relative protein levels in the normal group were normalized to 1.

### 2.10 Statistical analysis

All data were analysed using R version 4.3.0. Sample sizes (*n*) for animals, and the number of biological replicates for experiments are indicated in the corresponding figure legends. Values shown in the graphs are presented as means ± SEM. Statistical differences between groups were analysed using one‐way analysis of variance (ANOVA) statistical tests, with post‐hoc tests, or two‐tailed unpaired *t*‐tests with unequal variance. A *p*‐value <0.05 was considered statistically significant.

## 3 RESULTS

### 3.1 Farrerol exhibits binding affinity towards PTPN1

**FIGURE 1:**
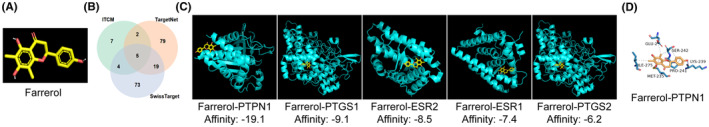
Farrerol bound to PTPN1. (A) 3D conformer of farrerol from PubChem. (B) Venn diagram showed the targets of farrerol by integrating ITCM, TargetNet, and SwissTarget databases. (C) Molecular docking between PTPN1 and five targets from (B) and the affinity of the ligand (PTPN1) and receptors (targets). (D) Visualization of the docking site.

To expedite the precise identification of the target of farrerol, we chose network pharmacology as our methodology. First, we obtained the 3D conformer (Figure 1A) and canonical SMILES (CC1 = C(C(=C2C(=C1O)C(=O)CC(O2)C3 = CC=C(C=C3)O)C)O) of farrerol (PubChem CID:442396) in the PubChem database. Next, based on its structure, we acquired five candidate (Figure 1B) targets of farrerol in vivo through the integration of ITCM, TargetNet and SwissTarget databases. These candidates were PTPN1, prostaglandin‐endoperoxide synthase 1 (PTGS1), oestrogen receptor 2 (ERS2), oestrogen receptor 1 (ERS1) and prostaglandin‐endoperoxide synthase 2 (PTGS2), respectively. Subsequently, we employed OpenBabel, PyMOL, AutoDock Vina software and RCSB PDB to perform molecular docking. PTPN1 was designated the ligand, while the targets served as the receptors. Furthermore, affinity calculations and visual presentations were conducted for the docking results (Figure 1C,D). Ultimately, we selected PTPN1 with the highest affinity to farrerol for further analysis and validation.

### 3.2 Farrerol regulates insulin signalling pathway through PTPN1

**FIGURE 2:**
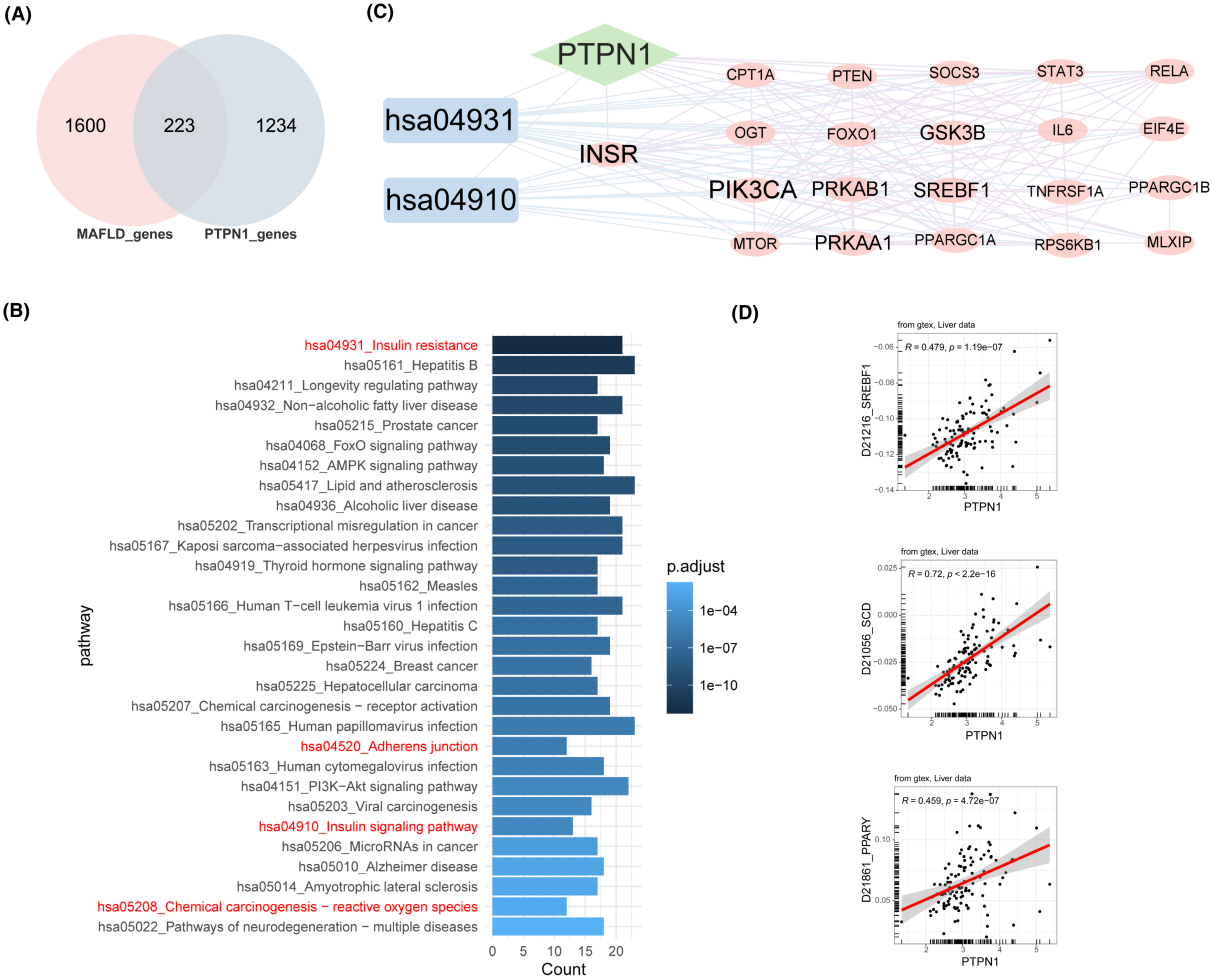
Farrerol regulates the insulin signalling pathway through PTPN1. (A) Venn diagram revealed 223 genes associated with MAFLD and PTPN1. (B) Top 30 pathways in the KEGG enrichment analysis of 223 genes. (C) Two PTPN1‐associated networks related to insulin resistance were determined using Cytoscape. (D) Correlation between PTPN1 and lipogenic genes in the liver (SREBF1, SCD and PPARγ).

To further investigate how farrerol prevents MAFLD by targeting PTPN1, we employed bioinformatics approaches for data mining. A total of 1823 MAFLD‐related disease genes were obtained from C0400966, C0015695, C4529962, C3241937 and C2711227 gene sets of the DisGeNET database (Table S1). Simultaneously, 1457 genes showed perturbations with PTPN1 in the liver, as seen from the GPSAdb database (Table S2). Using R software, we generated a Venn diagram to reveal 223 MAFLD‐related disease genes that exhibited perturbation relationships with PTPN1 (Figure 2A). To further study these 223 genes, KEGG enrichment analysis was performed using R (Table S3). Among the top 30 significant pathways in the enrichment analysis results, four pathways (hsa04931, hsa04520, hsa04910 and hsa05208) were associated with the PTPN1 gene (Figure 2B). In particular, two of these pathways were related to insulin resistance (hsa04931 and hsa04910). Subsequently, we visualized these two pathway networks using Cytoscape software, where the interaction relationships were derived from protein–protein interactions within the KEGG and STRING databases (Figure 2C). Furthermore, we validated the positive correlation between the mRNA expression levels of PTPN1 and SREBF1, SCD and PPARγ in the liver using data derived from the GTEx database (Figure 2D). Through the above analysis, we discovered that PTPN1 may potentially be involved in insulin resistance by regulating INSR phosphorylation, thereby influencing the transmission of the PI3K‐AKT signalling pathway and subsequently impacting the expression of downstream proteins related to lipid synthesis (SREBP1, SCD and PPARγ).

### 3.3 Farrerol alleviates HFD‐induced impaired glucose tolerance

**FIGURE 3:**
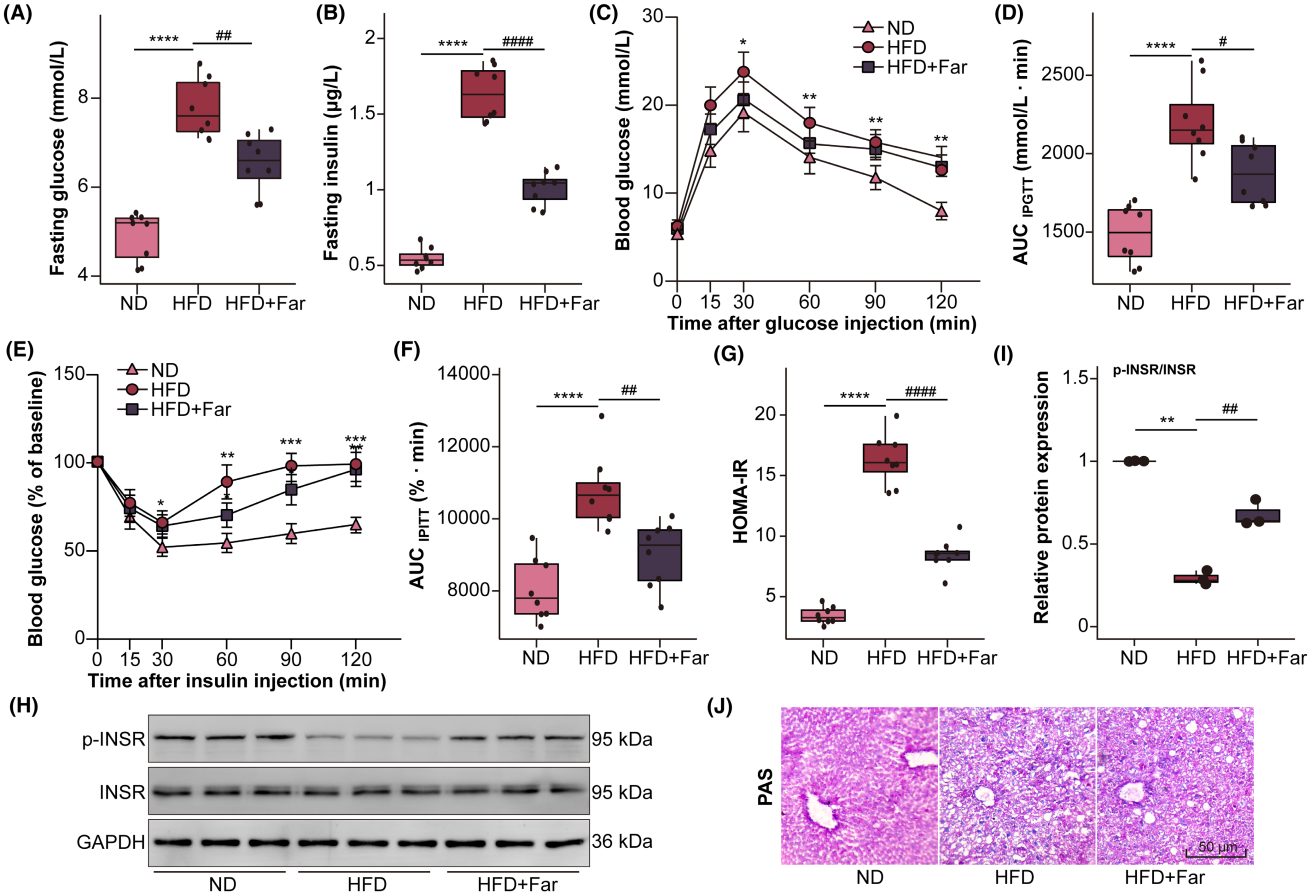
Effects of farrerol on glucose tolerance and insulin sensitivity. (A, B) Levels of blood glucose and insulin in mice after fasting for 12 h (*n* = 8). (C, D) Glucose tolerance tests (GTTs, 0.75 g/kg) and the calculation of the corresponding area under the curve (AUC) (*n* = 8). (E, F) Insulin tolerance tests (ITTs, 1.5 U/kg), presented as a percentage of baseline glucose to control for differences in baseline glucose, and the AUC calculations (*n* = 8). (G) HOMA‐IR was calculated based on (A) fasting glucose and (B) insulin levels (*n* = 8). (H, I) Expression of phospho‐INSR (p‐INSR), insulin receptor (INSR) and GAPDH was analysed by western blot. GAPDH served as a loading control (*n* = 3). *p < 0.05, **p < 0.01, ***p < 0.001, ^****^ *p* < 0.0001 versus ND group, ^#^ *p* < 0.05, ^##^ *p* < 0.01, ^###^ *p* < 0.001, ^####^ *p* < 0.0001 versus farrerol (Far) group. (J) Representative images of PAS staining of liver sections from mice (*n* = 8; scale bar, 50 μm).

Previous studies have shown that farrerol can alleviate liver injury, and we have demonstrated that farrerol has no significant toxic effect through the observation of liver tissue and stem cells (Figure S1). To further investigate the potential impact of farrerol on insulin resistance, we conducted GTTs and ITTs to evaluate glucose homoeostasis and insulin sensitivity in mice with or without farrerol supplementation. At baseline, both fasting glucose and insulin levels were elevated in mice fed a HFD compared to those on a ND. Notably, the HFD‐fed mice receiving farrerol supplementation showed lower levels of fasting glucose and insulin (Figure 3A,B). Furthermore, the impaired response to glucose load in the HFD‐fed mice was effectively restored following farrerol supplementation (Figure 3C,D), indicating its potential to improve glucose tolerance. Consistently, the HFD‐fed mice with farrerol supplementation exhibited a rapid response of glucose to insulin load (Figure 3E,F), along with a decreased HOMA‐IR score (Figure 3G). In addition, PTTs found that farrerol reduced Gluconeogenesis of HFD‐fed mice (Figure S3). Moreover, farrerol increased the expression of p‐INSR extracted from liver tissue, which was dephosphorylated in HFD‐fed mice (Figure 3H,I). However, liver glycogen content remained unaffected by farrerol (Figure 3J). Thus, farrerol supplementation significantly improves HFD‐induced insulin sensitivity and glucose tolerance in mice.

### 3.4 Farrerol inhibits hepatocyte steatosis through the PTPN1‐INSR signalling pathway

**FIGURE 4:**
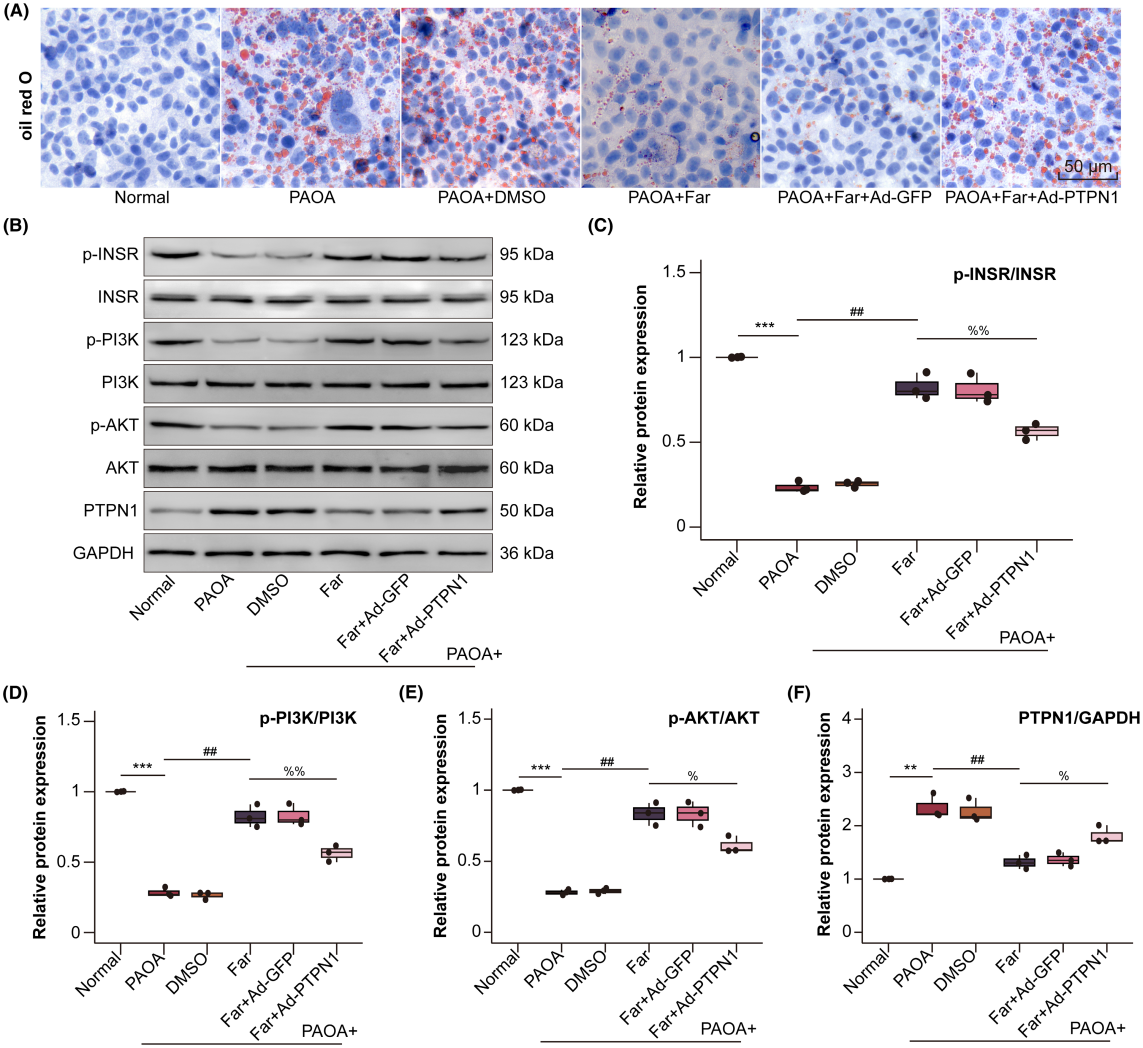
Farrerol reduces lipid accumulation in HepG2 cells treated with PAOA. (A) Representative oil red O staining images of HepG2 cells (scale bar, 50 μm. *n* = 3 independent experiments per group). (B–F) Expression of p‐INSR, INSR, p‐PI3K, PI3K, p‐AKT, AKT, PTPN1 and GAPDH was analysed using a western blot (the cells were stimulated with 100 nmol/L insulin for 10 min and then collected for western blot). **p < 0.05, ***p < 0.001 versus normal group, ^##^ *p* < 0.05 versus PAOA group, ^%^ *p* < 0.05, ^%%^ *p* < 0.01 versus far group.

To elucidate the precise mechanism underlying farrerol's impact on reducing lipid accumulation in hepatocytes, we conducted experiments using HepG2 cells. These cells were infected with an adenovirus carrying the PTPN1 gene, followed by treatment with PAOA. The results demonstrated that farrerol supplementation attenuated PAOA‐induced lipid accumulation in HepG2 cells (Figure 4A). Additionally, consistent with these findings, HepG2 cells overexpressing PTPN1 exhibited exacerbated lipid accumulation in response to PAOA stimulation compared to the control group. Subsequent immunoblotting analysis yielded results consistent with the aforementioned results, indicating that farrerol significantly increased the phosphorylation levels of INSR and PI3K‐AKT, thereby restoring the insulin signalling cascade. However, it is worth noting that this protective effect was abolished in the presence of PTPN1 overexpression (Figure 4B–G). Thus, farrerol exerts its ability to attenuate PAOA‐induced lipid droplet accumulation in HepG2 cells through its interaction with PTPN1.

### 3.5 Farrerol alleviates hepatic steatosis through PTPN1 in HFD‐fed mice

**FIGURE 5:**
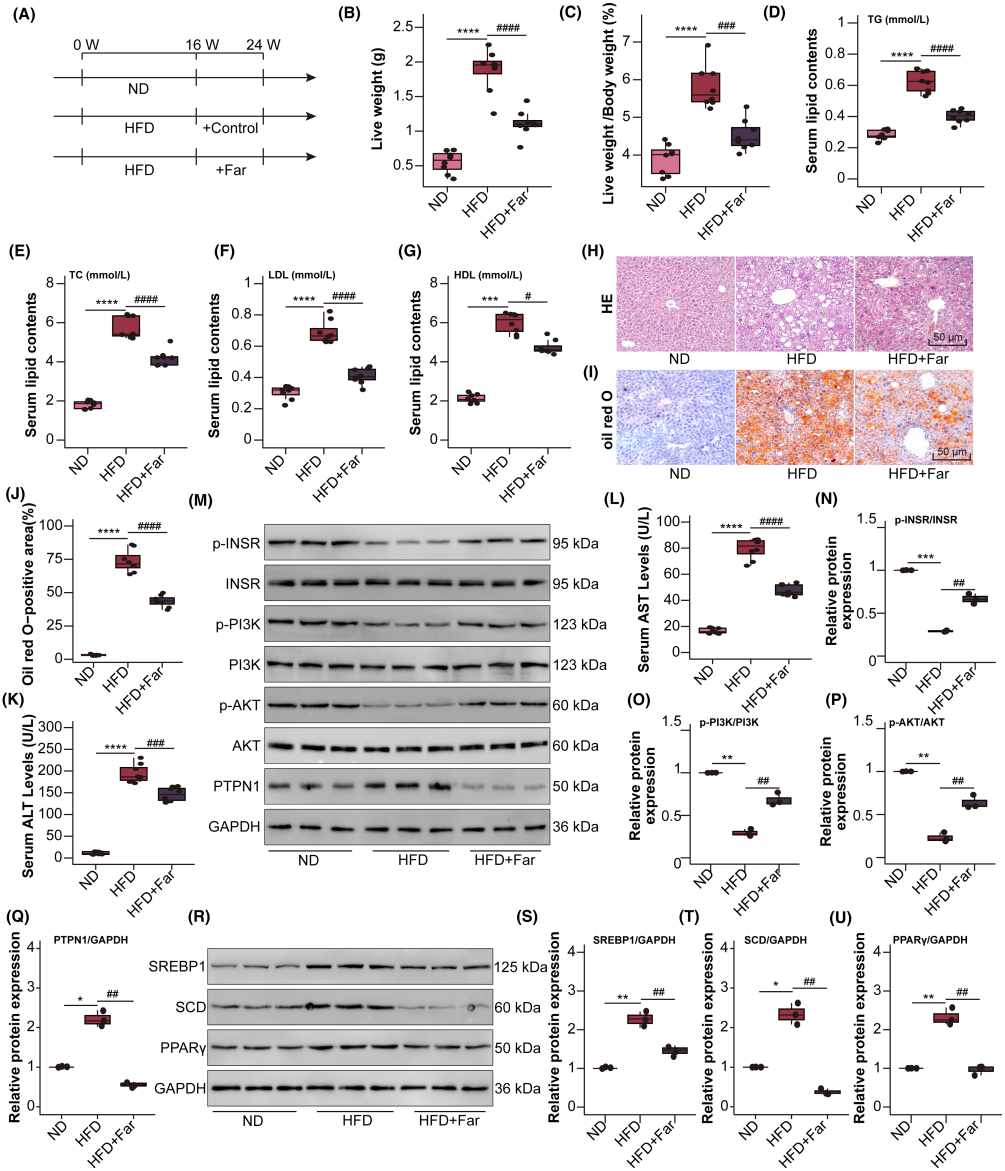
Farrerol alleviates hepatic steatosis through PTPN1 in HFD‐fed mice. (A) Schematic diagram of the experimental procedure (*n* = 8). (B, C) Liver weight and liver weight to body weight values of mice (*n* = 8). (D–G) Serum lipid (TG, TC, LDL and HDL) levels (*n* = 8). (H–J) Representative images of haematoxylin and eosin, and oil red O staining of liver sections from mice (*n* = 8; scale bar, 50 μm). (K, L) Serum levels of ALT and AST were measured in mice (*n* = 8). (M–U) Expression of p‐INSR, INSR, p‐PI3K, PI3K, p‐AKT, AKT, PTPN1, SREBP1, SCD, PPARγ and GAPDH was analysed using a western blot. *p < 0.05, **p < 0.05, ***p < 0.001, ****p < 0.0001 versus ND group, #*p* < 0.05, ##*p* < 0.01, ###*p* < 0.001, ####*p* < 0.0001 versus HFD group.

To examine the pharmacological effects of farrerol on hepatic steatosis and injury, we used an HFD‐induced MAFLD model, which was fed an HFD for 16 weeks and then administered farrerol i.p. for an additional 8 weeks with continuous HFD feeding (Figure 5A). Throughout the whole process of the experiment, the body weight of the HFD‐fed mice was significantly higher than that of the NDfed control mice. However, it is worth noting that farrerol had minimal effects on the body weight and food intake of mice on a normal diet (Figure S2). Moreover, the increased liver weight and liver weight‐to‐body weight ratio in the HFDfed mice were remarkably decreased by farrerol (Figure 5B,C). In addition, haematoxylin and eosin staining and oil red O staining showed that lipid accumulation in the HFD‐fed mice was significantly reduced by farrerol (Figure 5H–J), which was supported by the serum TG, TC, LDL and HDL levels (Figure 5D–G). The serum levels of ALT and AST were lower in farrerol‐treated mice than in the HFD‐fed mice (Figure 5K,L).

**FIGURE 6:**
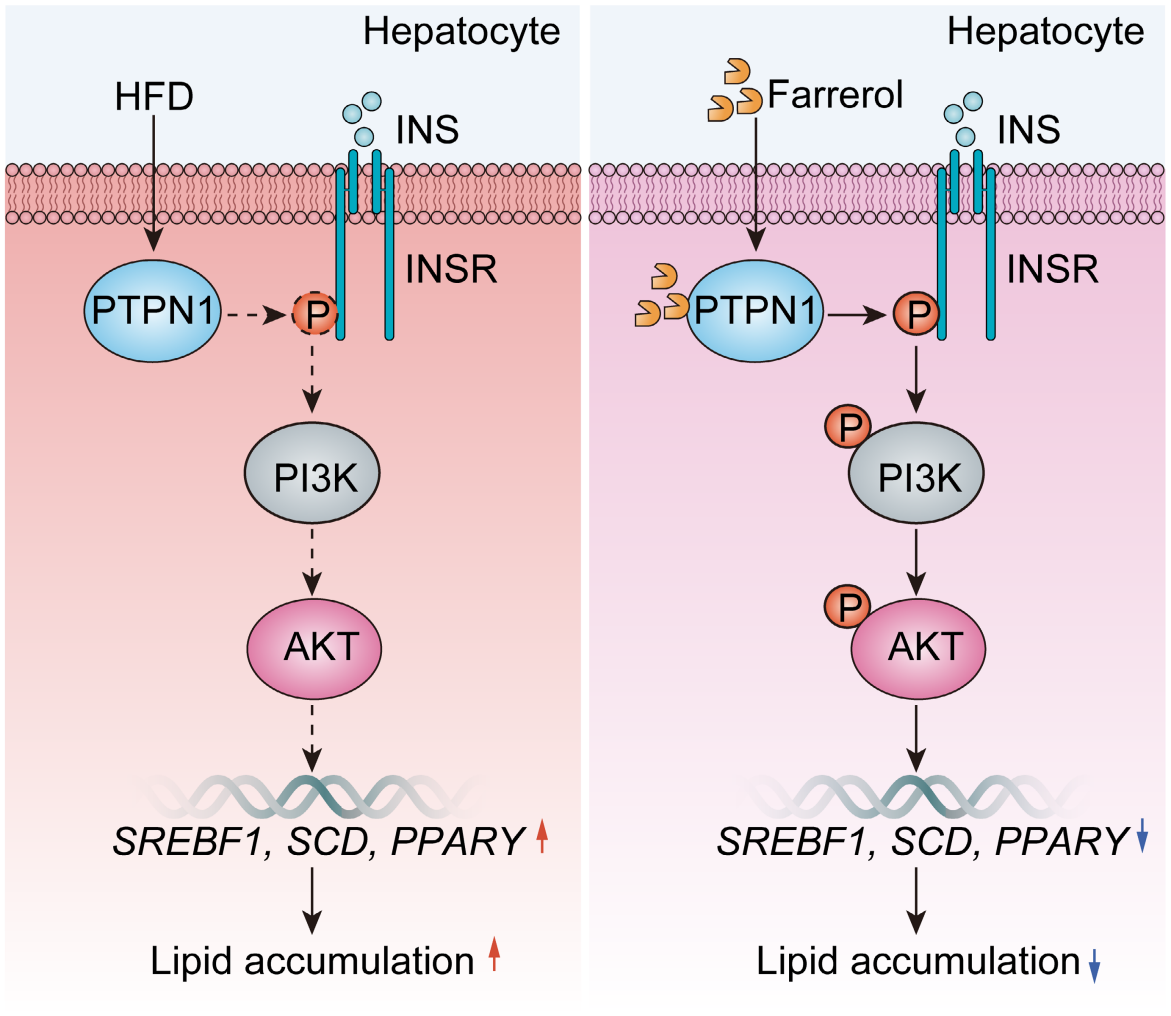
Schematic diagram of the protective function of farrerol against MAFLD via the activation of the PTPN1‐INSR pathway. Farrerol increases the phosphorylation of INSR via binding to PTPN1, leading to the activation of the PI3K/AKT signalling pathway and a decrease in lipogenic gene expression, which inhibits the IR and lipid accumulation, ultimately alleviating the development of MAFLD.

Moreover, the findings from western blot assays provided further evidence that farrerol effectively reversed the significant down‐regulation of p‐INSR, p‐PI3K and p‐AKT in mice fed an HFD due to increased PTPN1 expression (Figure 5N–Q). Furthermore, farrerol exhibited a notable inhibitory effect on the up‐regulation of adipogenic genes (SREBP1, SCD and PPARγ) in the HFD‐fed mice (Figure 5R–U). These results conclusively demonstrated that farrerol exerted a protective effect against hepatic steatosis and injury through its targeted modulation of the PTPN1‐INSR‐PI3K‐AKT signalling pathway (Figure 6).

## 4 DISCUSSION

Medicinal ingredients found in nature have great potential for treating diseases and may be safer than synthetic agents. Farrerol is natural flavonoid compound derived from the leaves of Ericaceae. Previous studies have shown it farrerol has protective effects on liver injury.^23^, ^37^ However, its function and mechanism in MAFLD are poorly understood. Network pharmacology analyzes the synergistic relationships among drugs, diseases, and targets by using network methods to understand how components interact with each other.^38^ According to the ITCM, TargetNet and Swisstarget databases, we identified five candidate genes as potential targets of farrerol in vivo. After affinity calculation and simulated molecular docking, PTPN1 was selected as the most potential target of farrerol. Combining MAFLD‐associated disease genes and PTPN1‐associated perturbator genes in liver, we identified 223 genes for KEGG enrichment analysis. Among the top 30 pathways, insulin resistance was top‐ranked and involved in the PTPN1‐INSR‐PI3K‐AKT signalling pathway.

PTPN1, a cytoplasmic protein tyrosine phosphatase, plays a crucial role in regulating various cellular signalling pathways through dephosphorylation, including the insulin signalling pathway. IR has been recognized as a key risk factor for MAFLD in previous research.^8^ In the context of insulin signal transduction, PTPN1 contributes to the inhibition of INSR activity by dephosphorylating its tyrosine residues, thereby serving as a negative feedback regulatory mechanism. Following the binding of insulin to its receptor, the receptor undergoes autophosphorylation, which activates downstream signalling cascades (such as PI3K and AKT). However, to maintain signalling homoeostasis and prevent excessive activation, phosphatases like PTPN1 are essential for dephosphorylating and terminating these signals.^39^ The association between PTPN1 and IR is further supported by various findings that link PTPN1 to IR and diabetes in humans.^40^ Mice with PTPN1 inhibition exhibited augmented phosphorylation of INSR and enhanced PI3K/AKT signalling, protecting against diet‐induced obesity, improving whole‐body glucose homoeostasis, decreasing TG and TC and diminishing expression of the lipogenic gene SREBP1.^12^, ^41^, ^42^, ^43^ PTPN1 has emerged as a therapeutic target in several human diseases and disorders, including MAFLD.^44^, ^45^ Despite substantial efforts, the development of highly selective PTPN1 inhibitors remains challenging due to the similarity among different phosphatases.^15^ In our study, farrerol exhibited significant affinity towards PTPN1 and improved insulin sensitivity and glucose tolerance, indicating its protective properties on MAFLD.

To verify the mechanism by which farrerol prevents MAFLD, we performed experiments in vitro and in vivo. Our study revealed that PTPN1 was highly expressed in PAOA‐induced hepG2 cells, which aligned with the findings reported in previous studies.^46^, ^47^ Farrerol treatment significantly restored the dephosphorylation of INSR by targeting PTPN1 and alleviated the steatosis of HepG2 cells by inhibiting the PI3K/AKT signalling pathway. In addition, farrerol reduced elevated liver weight, serum lipid content and aminotransferase, hepatic steatosis and lipid deposition in mice with HFD‐induced MAFLD. Similarly, the results of western blot assays further suggested that farrerol inhibited INSR dephosphorylation by binding to PTPN1 and downstream SREBP1, SCD and PPARγ activation, thereby postponing the pathogenesis of MAFLD.

MAFLD is regarded as a chronic hepatic condition associated with metabolic syndrome. It encompasses a spectrum of liver disorders, ranging from simple steatosis to steatohepatitis and liver fibrosis, with the latter two having the potential to progress to cirrhosis and hepatocellular carcinoma. Recent studies demonstrated that PTPN1 deficiency protected against liver inflammation and fibrosis,^48^, ^49^ and PTPN1 inhibitor exhibited protective effects on liver IR in equine metabolic syndrome. Therefore, we proceeded to further validate the role of farrerol in steatohepatitis and liver fibrosis.

Collectively, our current findings provide compelling evidence that farrerol, identified as a specific inhibitor of PTPN1, effectively ameliorates the pathogenesis of MAFLD. This is achieved through its ability to alleviate IR and suppress hepatic lipid accumulation. Therefore, our results not only contribute valuable insights towards the development of highly selective PTPN1 inhibitors but also offer promising therapeutic strategies for tackling MAFLD. Further investigations are warranted to explore the effects of farrerol on steatohepatitis and liver fibrosis.

## AUTHOR CONTRIBUTIONS

**Chunfang Xu:** Funding acquisition (equal); project administration (equal); supervision (equal); writing – review and editing (equal). **Jingwen Gao:** Conceptualization (equal); data curation (equal); investigation (equal); methodology (equal); resources (equal); writing – original draft (equal). **Xiaomin Cang:** Conceptualization (equal); data curation (equal); investigation (equal); methodology (equal); resources (equal); writing – original draft (equal). **Lu Liu:** Formal analysis (equal); methodology (equal). **Jiaxi Lin:** Software (equal); visualization (equal). **Shiqi Zhu:** Methodology (equal); software (equal). **Lihe Liu:** Investigation (equal); resources (equal). **Xiaolin Liu:** Project administration (equal); validation (equal). **Jinzhou Zhu:** Funding acquisition (equal); project administration (equal); supervision (equal).

## CONFLICT OF INTEREST STATEMENT

The authors confirm that there are no conflicts of interest.

## Supporting information


Data S1:

Data S2:

**Table S2:** 1457 genes showed perturbations with PTPN1 in the liver from the GPSAdb database.

**Table S3:** 223 MAFLD‐related disease genes that exhibited perturbation relationships with PTPN1.

## Data Availability

All data is available in the main text, the supplementary information files, or from the contact author, Dr. Chunfang Xu at xuchunfang@suda.edu.cn.
