# Supplementary material for: Farrerol alleviates insulin resistance and hepatic steatosis of metabolic associated fatty liver disease by targeting PTPN1
Source: J Cell Mol Med. 2024 Sep 17;28(18):e70096. doi: 10.1111/jcmm.70096 (PMC11408267; doi:10.1111/jcmm.70096)
Supplement: Supplementary file 1 — Data S1: [file JCMM-28-e70096-s004.docx]

**Farrerol alleviates insulin resistance and hepatic steatosis of metabolic associated fatty liver disease by targeting PTPN1**

Jingwen Gao ^a,b^ , Xiaomin Cang^c,1^, Lu Liu ^a,b^, Jiaxi Lin ^a,b^, Shiqi Zhu ^a,b^, Lihe Liu ^a,b^, Xiaolin Liu ^a,b^, Jinzhou Zhu ^a,b,*^, Chunfang Xu ^a,b,*^

**Supplementary figures**


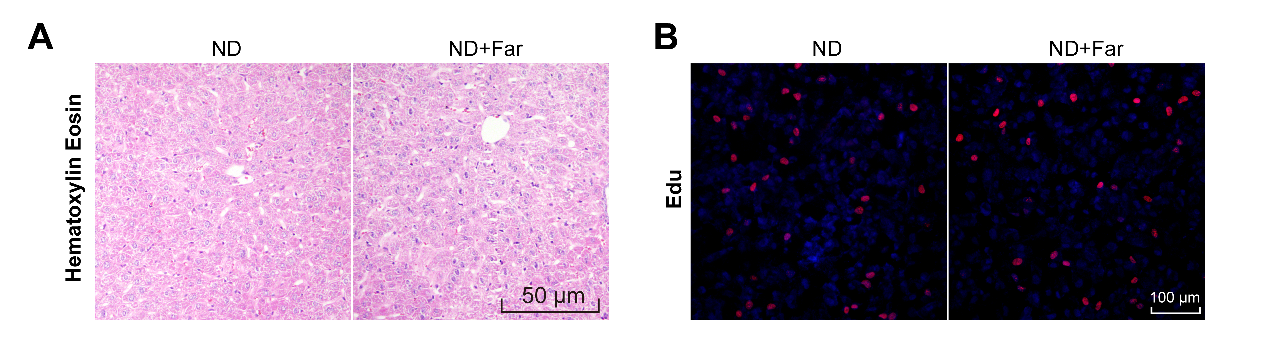


Supplementary Figure 1. Effect of Far on liver tissue and HepG2 cells. (A) Hematoxylin eosin staining on the liver from mice fed a normal diet and treated with Far. (B) Edu assays were performed on HepG2 cells treated with Far.


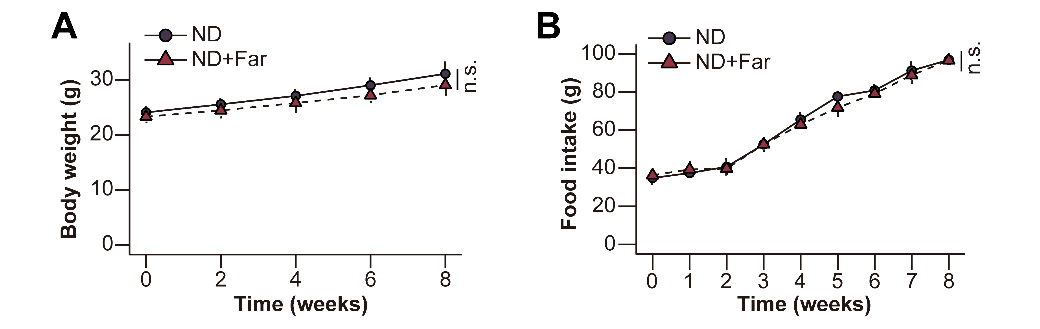


Supplementary Figure 2. Farrerol (Far) did not affect body weight or intake in ND mice. (A) Growth curve from 6-14 weeks of age (n = 8). (B) Food intake of 8 mice every week.


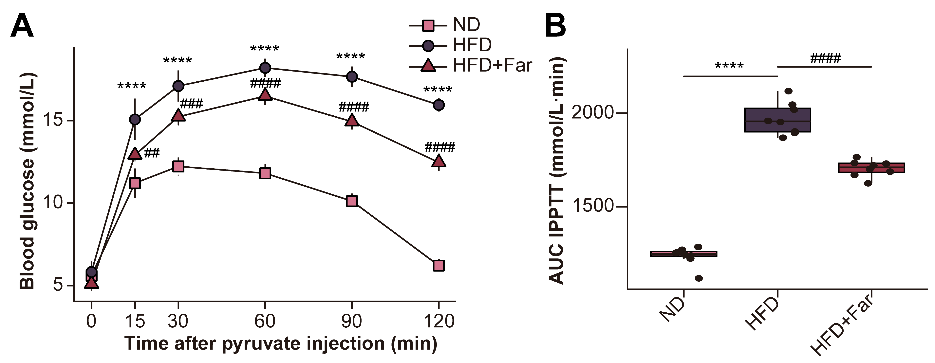


Supplementary Figure 3. Far reduced gluconeogenesis of HFD-induced mice. (A) PTT curve for glucose. (B) AUC for glucose in the PTT.

**Supplementary tables**

**Table S1** Antibodies used in this study.

| Antibody | Source | Catalog No. | Diluted ratio |
| --- | --- | --- | --- |
| INSR | Santa | sc-57344 | 1: 1000 |
| phospho-INSR | Santa | sc-534471 | 1: 1000 |
| phospho-PI3K | Cell Signaling | #17366 | 1: 1000 |
| PI3K/AKT set | Abcam | ab283852 |  |
| PI3K | Abcam | ab191606 | 1:2000 |
| AKT | Abcam | ab179463 | 1:10000 |
| phospho-AKT | Abcam | ab192623 | 1:1000 |
| PTPN1 | Proteintech | 11334-1-AP | 1:5000 |
| SREBF1 | Proteintech | 66875-1-AP | 1:1000 |
| SCD | Proteintech | 28678-1-AP | 1:2000 |
| PPARG | Proteintech | 16643-1-AP | 1:1000 |
